# Full-Duplex Relay with Delayed CSI Elevates the SDoF of the MIMO X Channel

**DOI:** 10.3390/e23111484

**Published:** 2021-11-10

**Authors:** Tong Zhang, Gaojie Chen, Shuai Wang, Rui Wang

**Affiliations:** 1 The Department of Electrical and Electronic Engineering, Southern University of Science and Technology, Shenzhen 518055, China; zhangt7@sustech.edu.cn (T.Z.); wangs3@sustech.edu.cn (S.W.); 2 5GIC & 6GIC, Institute for Communication Systems, University of Surrey, Guildford GU2 7XH, UK; gaojie.chen@surrey.ac.uk

**Keywords:** delayed CSIT, information-theoretical security, MIMO X channel, MIMO relay, secure degrees-of-freedom

## Abstract

In this article, the sum secure degrees-of-freedom (SDoF) of the multiple-input multiple-output (MIMO) X channel with confidential messages (XCCM) and arbitrary antenna configurations is studied, where there is no channel state information (CSI) at two transmitters and only delayed CSI at a multiple-antenna, full-duplex, and decode-and-forward relay. We aim at establishing the sum-SDoF lower and upper bounds. For the sum-SDoF lower bound, we design three relay-aided transmission schemes, namely, the relay-aided jamming scheme, the relay-aided jamming and one-receiver interference alignment scheme, and the relay-aided jamming and two-receiver interference alignment scheme, each corresponding to one case of antenna configurations. Moreover, the security and decoding of each scheme are analyzed. The sum-SDoF upper bound is proposed by means of the existing SDoF region of two-user MIMO broadcast channel with confidential messages (BCCM) and delayed channel state information at the transmitter (CSIT). As a result, the sum-SDoF lower and upper bounds are derived, and the sum-SDoF is characterized when the relay has sufficiently large antennas. Furthermore, even assuming no CSI at two transmitters, our results show that a multiple-antenna full-duplex relay with delayed CSI can elevate the sum-SDoF of the MIMO XCCM. This is corroborated by the fact that the derived sum-SDoF lower bound can be greater than the sum-SDoF of the MIMO XCCM with output feedback and delayed CSIT.

## 1. Introduction

The deployment of 5G and all of the connections to 6G around the world have all exacerbated the concerns for information-theoretic security in mobile communication networks [1,2,3]. The secure degrees-of-freedom (SDoF) of multiple-input multiple-output (MIMO) networks with confidential messages and perfect channel state information at the transmitter (CSIT) was studied in [4,5,6,7,8,9]. For the *K*-user single-input single-output (SISO) interference channel with confidential messages (ICCM), the sum-SDoF was characterized in [4]. For the multiple-input multiple-output (MIMO) ICCM, under symmetric antenna configurations, the sum-SDoF was derived in [5,6]. Then, the sum-SDoF of the MIMO ICCM with arbitrary antenna configurations was characterized in [7]. The X network with confidential messages has a more general message setting than that in ICCM. For the X network with confidential messages, the sum-SDoF was studied in [8]. The sum-SDoF of rank-deficient ICCM and broadcast channel with confidential messages (BCCM) was characterized in [9].

For the fast-fading wireless channel, the CSIT can be delayed, that is, mismatching with current CSI but matching with past CSI. Under this imperfect setting, i.e., delayed CSIT, the research of SDoF was stemmed from [10], where under arbitrary antenna configurations, the SDoF region of two-user MIMO BCCM and delayed CSIT was characterized. Thereafter, the SDoF regions of two-user multiple-input single-output (MISO) BCCM with alternating CSIT was derived in [11]. The linear SDoF of blind multi-user MISO wiretap channel with delayed CSIT was characterized in [12]. Recently, in [13], the interplay of link connectivity and alternating CSIT in MISO BCCM was studied from the SDoF perspective. Aside from the BCCM, there are many efforts devoted to investigate the SDoF of two-user interference networks with delayed feedback [14,15,16,17]. In [14], under symmetric antenna configurations, a sum-SDoF lower bound for MIMO ICCM with delayed CSIT was obtained. In [15], under symmetric antenna configurations, a higher sum-SDoF lower bound than that in [14] was derived for MIMO XCCM with delayed CSIT, considering two more confidential messages. In [16], under symmetric antenna configurations, the sum-SDoF of MIMO ICCM with local output feedback was studied. In [17], under symmetric antenna configurations, the SDoF region of MIMO XCCM with output feedback and delayed CSIT was characterized, which has the highest sum-SDoF over that in [14,15,16].

Even with delayed CSI, it is shown in [18,19,20,21,22] that the decode-and-forward relay (for brevity, we henceforth use “relay” to stand for “decode-and-forward relay”) can assist the communication and enhance the degrees-of-freedom (DoF) of MIMO networks. When there is no CSIT, reference [19,20,21,22] considered the assistance of the relay with delayed CSI. In [19], a multiple-antenna relay with delayed CSI elevated the DoF of *K*-user SISO interference channel. In [21], a multiple-antenna relay with delayed CSI elevated the DoF of the SISO X channel. For the MIMO X channel, it is shown in [22] that a multiple-antenna relay with delayed CSI was able to enhance the DoF. As for X networks, *L* multiple-antenna full-duplex relays enhanced the sum-DoF of the 2×K X networks, as shown in [20]. Furthermore, having the security constraints, only the work in [23] addressed the sum-SDoF characterization of 2×2×2 SISO ICCM with delayed CSIT.

However, to summarize, none of existing works considered the SDoF of the relay-aided MIMO XCCM, where there is no CSI at two transmitters and a delayed CSI at the relay, such as the CSI model in [20,22]. For the first time, we consider the SDoF problem of such a system. Specifically, we consider a multiple-antenna full-duplex relay with delayed CSI in the MIMO XCCM with arbitrary antenna configurations, where there is no CSI at two transmitters. However, there are two major challenges. The first one is how to design a transmission scheme with security guarantee and transmission efficiency. The second one is how to create an upper bound for the sum-SDoF in a non-trivial way. We address these two problems in the following manner: We propose the relayed-aided jamming and interference alignment (IA) design for transmission scheme, which simultaneously fulfills security guarantee and transmission efficiency. In addition, we leverage the existing SDoF region of MIMO BCCM to design a sum-SDoF upper bound. The sum-SDoF lower and upper bounds are tight for partial antenna configurations, and thus, the sum-SDoF is derived therein. The main contributions of this paper are summarized as follows:

- To derive the sum-SDoF lower bound, under arbitrary antenna configurations, we propose three relay-aided transmission schemes, whose achievable sum-SDoF serves as the sum-SDoF lower bound. Specifically, we propose the relay-aided jamming scheme, and the relay-aided jamming and one-receiver IA scheme, and the relay-aided jamming and two-receiver IA scheme, where each scheme corresponds to one case of antenna configurations. In each scheme, the security and decoding are analyzed.
- To obtain the sum-SDoF upper bound, which does not appear in the existing literature and is non-trivial, we first treat two transmitters and the relay as a co-located transmitter, which is an enhanced scenario. Thereafter, we apply the results of the existing SDoF region of two-user MIMO BCCM, which is proven in [10], into this enhanced scenario.
- Our results show that if the full-duplex relay has double antennas of the receiver, the proposed sum-SDoF lower bound is not less than the sum-SDoF in existing two-user interference networks with delayed feedback in [14,15,16,17], and can be higher than the sum-SDoF of MIMO X channel with output feedback and delayed CSIT in [17]. Moreover, the proposed sum-SDoF lower bound matches with the sum-SDoF upper bound for partial antenna configurations, characterizing the sum-SDoF for these antenna configurations.

*Notations*: The identity matrix of dimensions *m* is denoted by $I_{m}$. The block-diagonal matrix with blocks A and B is denoted by BD{A,B}. The rank of matrix A is denoted by RKA. The log is referred to $\log_{2}$. ${\left[x\right]}^{+}=\max\left\{x,0\right\}$.

*Organizations*: The rest of this paper is organized as follows: The system model is defined in Section 2. The main results and discussions are presented in Section 3. We prove the Theorem 1 in Section 4. We draw our conclusions in Section 5.

## 2. System Model

**Figure 1:**
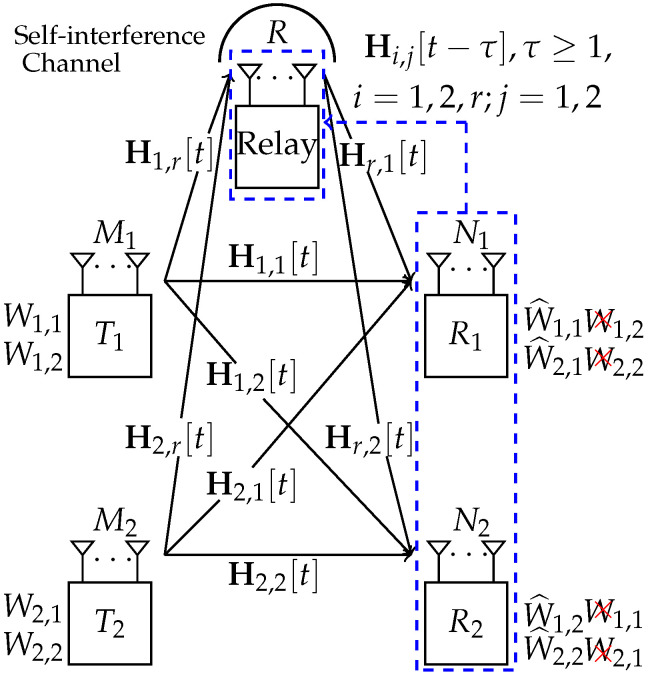
Illustration of the scenario, where there are delayed CSI at the relay and no CSI at two transmitters.

We consider the MIMO XCCM aided by a *R*-antennas full-duplex (the experimental studies and prototypes on full-duplex techniques can be found in [24,25,26,27,28,29], where the self-interference can be suppressed by radio frequency, analogue, and digital cancellations to achieve the noise floor; the details of self-interference suppression are out of the scope of this work) relay, where two transmitters (denoted by $T_{1}$ and $T_{2}$) have $M_{1}$ and $M_{2}$ antennas, respectively, and two receivers (denoted by $R_{1}$ and $R_{2}$) have $N_{1}$ and $N_{2}$ antennas, respectively, as shown in Figure 1. Namely, the antenna configurations are arbitrary. Without loss of generality, we have $N_{1}\leq N_{2}$ by denoting the receiver with most antennas by $R_{2}$. The $T_{i}$ has a confidential message $W_{i,j}$ for $R_{j}$, where i,j=1,2. The CSI matrix from $T_{1},T_{2}$ and the relay to $R_{1},R_{2}$ and the relay at time slot *t* is denoted by $H_{i,j}\left[t\right],i,j=1,2,r$, respectively, where $H_{i,j}\left[t\right]$ is time-varying and independently distributed across space and time. There is no CSI at two transmitters. At the time slot *t*, both $R_{1}$ and $R_{2}$ have the instantaneous knowledge of $H_{i,1}$ and $H_{i,2}$, i=1,2,r; the relay has the instantaneous knowledge of $H_{i,r},i=1,2$. Moreover, due to feedback delay (the CSI feedback link is assumed to be additional, compared with the data/artificial noise transmission link), the past CSI matrices $H^{t-1}≜\left\{H_{i,j}\left[t-\tau \right],t>\tau \geq 1,i=1,2,r;j=1,2\right\}$ are available at the relay. Note that the definition of CSI in this paper is the same as that defined in [21,22].

A code $\left\{2^{nR_{i,j}\left(\eta \right)},i,j=1,2\right\}$ with achievable secrecy rates $R_{i,j}\left(\eta \right),i,j=1,2$ is defined below, where η denotes the signal-to-noise ratio (SNR). The communication process takes *n* channel uses (time slots) with confidential messages $W_{i,j}=\left[1,\cdots ,2^{nR_{i,j}\left(\eta \right)}\right]$ (from $T_{i}$ to $R_{j}$). At the time slot *t*, a stochastic encoder $f_{i,j}\left(\cdot \right)$ at the $T_{i}$ encodes confidential message $W_{i,j}$ to an input signal $x_{i,j}\left[t\right]$, i.e., $x_{i,j}\left[t\right]=f_{i,j}\left(W_{i,j}\right)$. At the time slot *t*, a stochastic encoder $f_{r}\left(\cdot \right)$ at the full-duplex relay encodes the collection of received signals $y_{r}^{t}≜\left[y_{r}\left[1\right],\cdots ,y_{r}\left[t\right]\right]$ and delayed CSI $H^{t-1}$ to an input signal $x_{r,j}^{t}$, i.e., $x_{r,j}\left[t\right]=f_{r}\left(y_{r}^{t},H^{t-1}\right)$. At the time slot *n*, a decoder $g_{i,j}\left(\cdot \right)$ at the $R_{j}$ decodes the output signals $y_{j}^{n}$ and CSI matrices $H^{n}$ to an estimated message ${\hat{W}}_{i,j}$, i.e., ${\hat{W}}_{i,j}=g_{i,j}\left(y_{j}^{n},H^{n}\right)$. According to [10], the secure code satisfies the reliability criterion,

(1) $$\mathrm{Pr}\left[W_{i,j}\neq {\hat{W}}_{i,j}\right]\leq \epsilon_{n},\;i,j=1,2,$$

and the weak secrecy criterion,

(2a) $$\begin{array}{ccc} & & \frac{1}{n}I\left(W_{i,1};y_{2}^{n}\right)\leq \epsilon_{n}, \end{array}$$

(2b) $$\begin{array}{ccc} & & \frac{1}{n}I\left(W_{i,2};y_{1}^{n}\right)\leq \epsilon_{n},\;i=1,2, \end{array}$$

where $\epsilon_{n}\to 0$ as n→1.

The sum of the secure channel capacity is defined as $C_{s}=\max\sum_{i=1}^{2}\sum_{j=1}^{2}R_{i,j}\left(\eta \right).$ The sum-SDoF is a first-order approximation of the secure sum-capacity in the high SNR regime and defined as follows:

(3) $$\sum_{i=1}^{2}\sum_{j=1}^{2}d_{i,j}=\lim_{\eta \to 1}\frac{C_{s}}{\log\eta }.$$

## 3. Main Results and Discussions

**Theorem** **1.** ***(sum-SDoF lower bound under arbitrary antenna configurations)**. For the relay-aided MIMO XCCM with delayed CSI at the multiple-antenna full-duplex relay and no CSI at two transmitters, the sum-SDoF lower bound under arbitrary antenna configurations is given as follows:*

(4) $$\begin{array}{c} \sum_{i=1}^{2}\sum_{j=1}^{2}d_{i,j}\geq \left\{\begin{array}{cc} \frac{\left(M_{1}+M_{2}\right)\left(x_{1}+x_{2}\right)}{1+x_{1}+x_{2}}, & M_{1}+M_{2}\leq N_{1}\;\;\&\;\;N_{2}<R,\\ \frac{\min\left\{M_{1}+M_{2},R\right\}x_{1}+\left(M_{1}+M_{2}\right)x_{2}}{1+x_{1}+x_{2}+x_{3}}, & N_{1}<M_{1}+M_{2}\leq N_{2}\;\;\&\;\;N_{2}<R,\\ \frac{\left(M_{1}+M_{2}\right)x_{1}}{1+x_{1}}, & M_{1}+M_{2}\leq N_{2}\;\;\&\;\;N_{1}<R\leq N_{2},\\ \frac{\min\left\{M_{1}+M_{2},R,N_{1}+N_{2}\right\}\left(x_{1}+x_{2}\right)}{1+x_{1}+x_{2}+\max\left\{x_{4},x_{5}\right\}}, & N_{2}<M_{1}+M_{2}\;\;\&\;\;N_{2}<R,\\ \frac{\min\left\{M_{1}+M_{2},R,N_{1}+N_{2}\right\}x_{1}}{1+x_{1}+x_{5}}, & N_{2}<M_{1}+M_{2}\;\;\&\;\;N_{1}<R\leq N_{2},\\ 0, & R\leq N_{1}, \end{array}\right. \end{array}$$

*where*

(5a) $$\begin{array}{ccc} & & x_{1}=\frac{\min\{N_{2},R-N_{1}\}}{N_{1}}, \end{array}$$

(5b) $$\begin{array}{ccc} & & x_{2}=\frac{\min\{N_{1},R-N_{2}\}}{N_{2}}, \end{array}$$

(5c) $$\begin{array}{ccc} & & x_{3}=\frac{\min\left\{M_{1}+M_{2}-N_{1},R-N_{1}\right\}\min\left\{N_{1},R-N_{2}\right\}}{\min\left\{N_{1},R\right\}N_{2}}, \end{array}$$

(5d) $$\begin{array}{ccc} & & x_{4}=\frac{\min\left\{M_{1}+M_{2}-N_{1},N_{2},R-N_{1}\right\}\min\left\{N_{1},R-N_{2}\right\}}{\min\left\{N_{1},R\right\}N_{2}}, \end{array}$$

(5e) $$\begin{array}{ccc} & & x_{5}=\frac{\min\left\{M_{1}+M_{2}-N_{2},N_{1},R-N_{2}\right\}\min\left\{N_{2},R-N_{1}\right\}}{\min\left\{N_{1},R\right\}N_{1}}. \end{array}$$

**Proof.**  Please refer to Section 4. □

**Remark** **1.** ***(sum-SDoF lower bound under symmetric antenna configurations)**. For the relay-aided MIMO XCCM with delayed CSI at the multiple-antenna full-duplex relay and no CSI at two transmitters, the sum-SDoF lower bound under symmetric antenna configurations is given as follows by setting $N_{1}=N_{2}=N$ and $M_{1}=M_{2}=M$ in Theorem 1:*

(6) $$\sum_{i=1}^{2}\sum_{j=1}^{2}d_{i,j}\geq \left\{\begin{array}{cc} \frac{4M}{3}, & 2M<N\;\;\&\;\;2N<R,\\ \frac{4M(R-N)}{2R-N}, & 2M<N\;\;\&\;\;N<R\leq 2N,\\ \frac{2N\min\{M,N\}}{\min\{M+N,2N\}}, & N\leq 2M\;\;\&\;\;2N<R,\\ \frac{2N\min\{2M,R\}(R-N)}{N^{2}+\min\left\{2M+N,R+N\right\}\left(R-N\right)}, & N\leq 2M\;\;\&\;\;N<R\leq 2N,\\ 0, & R\leq N. \end{array}\right.$$

**Proposition** **1.** ***(sum-SDoF upper bound under arbitrary antenna configurations)**. For the relay-aided MIMO XCCM with delayed CSI at the multiple-antenna full-duplex relay and no CSI at two transmitters, the sum-SDoF upper bound under arbitrary antenna configurations is given by the following:*

(7) $$\sum_{i=1}^{2}\sum_{j=1}^{2}d_{i,j}\leq \frac{N_{1}\min\left\{M_{1}+M_{2}+R-N_{2},N_{1}\right\}+N_{2}\min\left\{M_{1}+M_{2}+R-N_{1},N_{2}\right\}}{\min\{M_{1}+M_{2}+R,N_{1}+N_{2}\}}.$$

**Proof.**  We enhance the channel by supposing that the two transmitters and the relay constitute a co-located transmitter. Based on ([10], Theorem 3), Proposition 1 is proven. □

**Proposition** **2.** ***(antenna configurations for sum-SDoF characterization)**. For the relay-aided MIMO XCCM with delayed CSI at the multiple-antenna full-duplex relay and no CSI at two transmitters, the sum-SDoF is characterized by the following:*

(8) $$\sum_{i=1}^{2}\sum_{j=1}^{2}d_{i,j}=N,\;N\leq M\;\;\&\;\;2N\leq R$$

**Proof.**  Following Theorem 1 and Proposition 1, we can verify the match of the sum-SDoF upper and lower bounds for N≤M and 2N≤R. Therefore, the value of sum-SDoF is *N* for N≤M and 2N≤R. □

**Figure 2:**
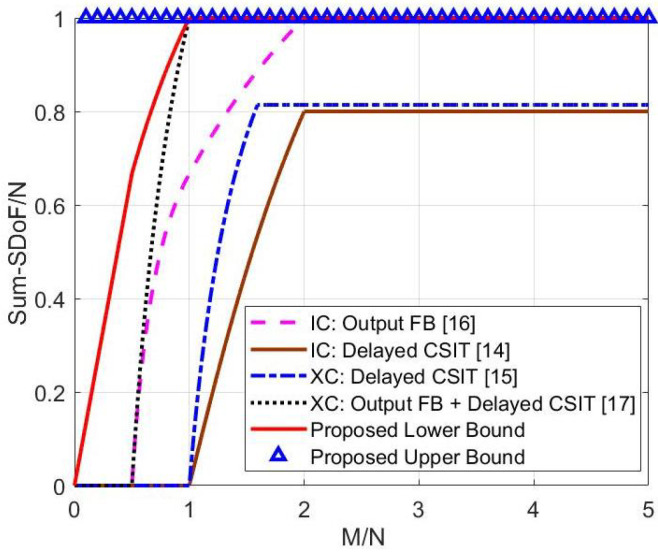
SDoF comparison with existing results [14,15,16,17] when 2N≤R.

**Remark** **2.** ***(advantages over existing results in two-user MIMO interference networks with delayed feedback [14,15,16,17])**: Under symmetric antenna configurations, the advantages of the derived results over existing results in two-user MIMO interference networks with delayed feedback [14,15,16,17] are shown in Figure 2. In particular, Figure 2 shows that if 2N≤R, the derived sum-SDoF lower bound is higher than the sum-SDoF of MIMO XCCM with output feedback and delayed CSIT in [17] for M<N, the sum-SDoF of MIMO ICCM with local output feedback in [16] for M<2N, the sum-SDoF lower bound of MIMO XCCM with delayed CSIT in [15] and the sum-SDoF lower bound of MIMO ICCM with delayed CSIT in [14] for all antenna configurations. The gain of our lower bound comes from the full-duplex relay taking over the artificial noise and interference transmission from the two transmitters.*

**Figure 3:**
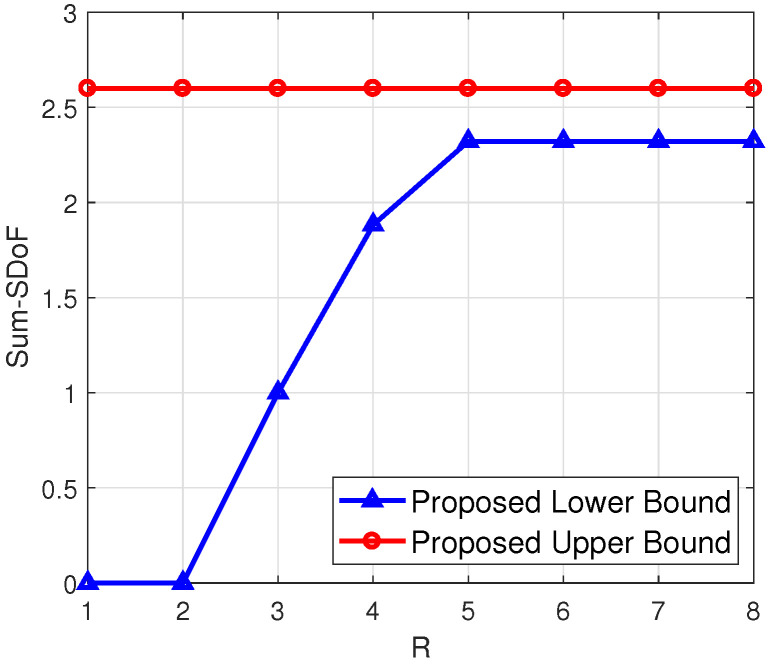
$\left(N_{1},N_{2},M_{1},M_{2}\right)=\left(2,3,2,3\right)$.

**Figure 4:**
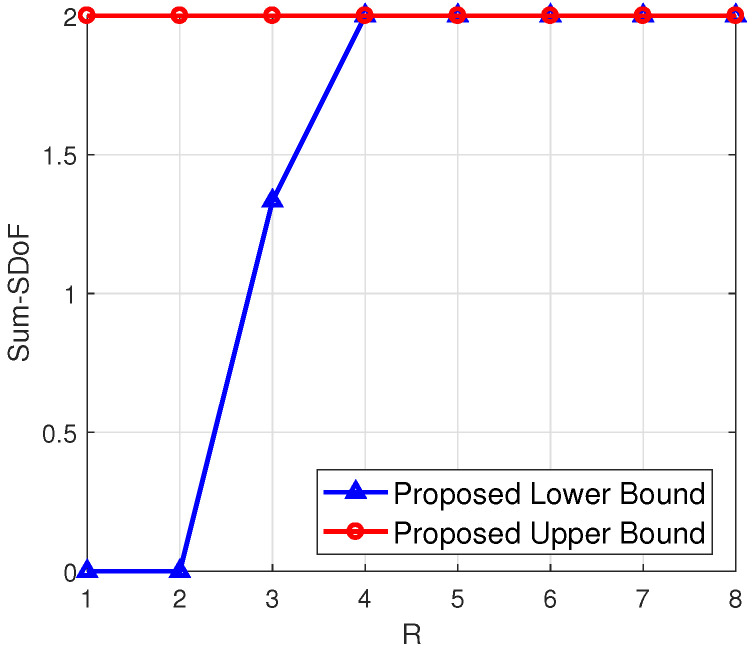
$\left(N_{1},N_{2},M_{1},M_{2}\right)=\left(2,2,2,3\right)$.

**Figure 5:**
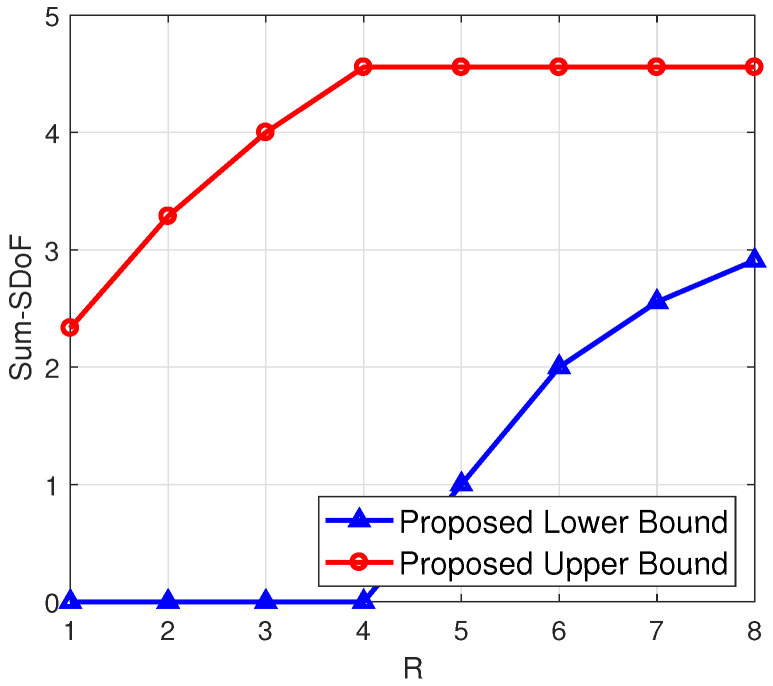
$\left(N_{1},N_{2},M_{1},M_{2}\right)=\left(4,5,2,3\right)$.

**Figure 6:**
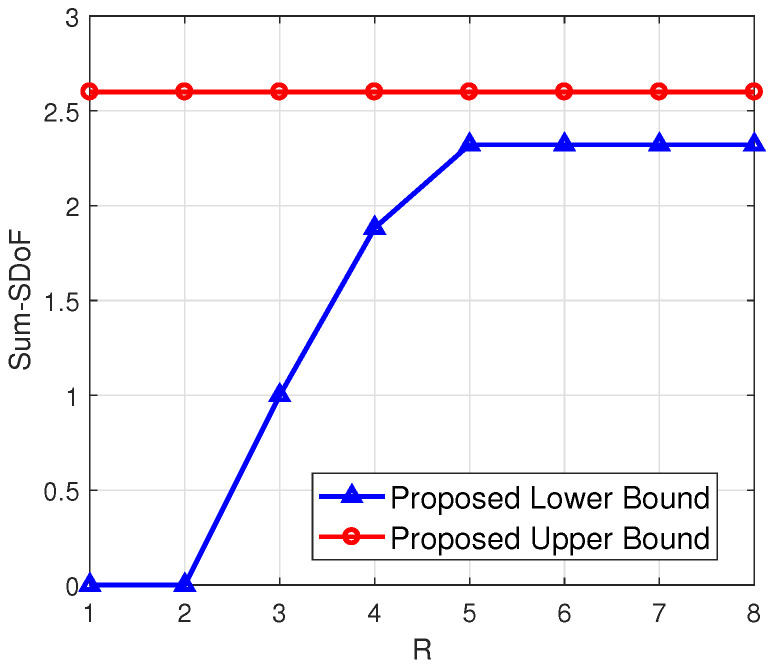
$\left(N_{1},N_{2},M_{1},M_{2}\right)=\left(2,3,4,5\right)$.

**Remark** **3.** ***(numerical calculation under arbitrary antenna configurations)**. To illustrate the proposed upper and lower bounds for arbitrary antenna configurations, we have shown four examples in Figure 3, Figure 4, Figure 5 and Figure 6, respectively. From Figure 3 and Figure 4, it can be seen that the smaller difference between $N_{1}$ and $N_{2}$ leads to the smaller gap between the proposed upper and lower bounds, when $M_{1},M_{2}$ are fixed and R is sufficiently large. Additionally, a higher $N_{2}$ leads to a higher value of the proposed lower bound, when $N_{1},M_{1},M_{2}$ are fixed. From Figure 5 and Figure 6, it can be seen that when $N_{1}+N_{2}$ is less than $M_{1}+M_{2}$ and R is sufficiently large, the gap between the proposed upper and lower bounds becomes smaller.*

## 4. Proof of Theorem 1: Relay-Aided Transmission Scheme Design

### 4.1. $M_{1}+M_{2}\leq N_{1}$ Case: The Relay-Aided Jamming Scheme

**Figure 7:**
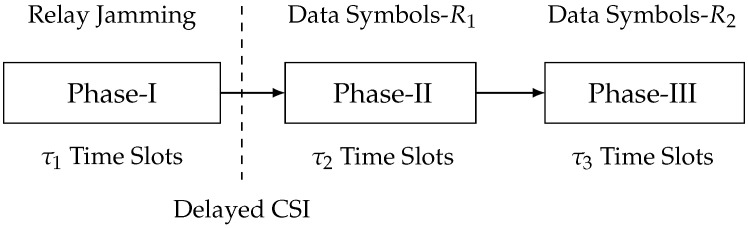
Flowchart of the relay-aided jamming scheme.

When $M_{1}+M_{2}\leq N_{1}$, each receiver can immediately decode the transmitted data symbols, which implies there is no need for IA. At the same time, the relay can send artificial noise symbols to ensure secure data symbol transmission. Therefore, we propose a relay-aided jamming scheme with three phases, where the relay cooperates with two transmitters for artificial noise transmission. In Phase I, the relay sends artificial noise symbols. In Phase II, two transmitters send data symbols for $R_{1}$; meanwhile, the output signal of Phase I at $R_{1}$ is reconstructed and re-transmitted by relay. In Phase III, two transmitters send data symbols for $R_{2}$; meanwhile, the output signal of Phase I at $R_{2}$ is reconstructed and re-transmitted by relay. The flowchart of this scheme is illustrated in Figure 7. Firstly, we define the holistic CSI matrices for this scheme as follows:

$$\begin{array}{ccc} & & H_{i,j}^{P\text{-}I}≜\mathrm{BD}\left\{H_{i,j}\left[1\right],\cdots ,H_{i,j}\left[\tau_{1}\right]\right\},\\ & & H_{i,j}^{P\text{-}\mathrm{II}}≜\mathrm{BD}\left\{H_{i,j}\left[\tau_{1}+1\right],\cdots ,H_{i,j}\left[\tau_{1}+\tau_{2}\right]\right\},\\ & & H_{i,j}^{P\text{-}\mathrm{III}}≜\mathrm{BD}\left\{H_{i,j}\left[\tau_{1}+\tau_{2}+1\right],\cdots ,H_{i,j}\left[\tau_{1}+\tau_{2}+\tau_{3}\right]\right\}, \end{array}$$

where i,j=1,2,r, and the value of $\tau_{1}$, $\tau_{2}$, and $\tau_{3}$ is determined based on the security analysis of the scheme. Moreover, we introduce the pre-stored full-rank matrices $\Phi_{1}\in C^{R\tau_{2}\times N_{1}\tau_{1}}$ and $\Phi_{2}\in C^{R\tau_{3}\times N_{2}\tau_{1}}$, whose items follow complex Gaussian CN(0,1). Next, the proposed three-phase transmission scheme and related analysis are elaborated.

*Phase I (Jamming by the Relay)*: This phase aims at sending $R\tau_{1}$ artificial noise symbols from the relay in $\tau_{1}$ time slots by *R* antennas. Denote the artificial noise vector sent by *R* antennas in $\tau_{1}$ time slots as $u\in C^{R\tau_{1}}$. The received signals of Phase-I at two receivers are expressed as follows:

(9) $$y_{j}^{P\text{-}I}=H_{r,j}^{P\text{-}I}u,\;j=1,2.$$

Note that additive white Gaussian noise (AWGN) is omitted, due to the SDoF analysis. The CSI matrices of Phase I return to the relay at the end of Phase I.

*Phase II (joint data transmission for $R_{1}$ by two transmitters and Phase I output transmission by the relay)*: We aim at sending $M_{1}\tau_{2}$ data symbols to $R_{1}$ from $T_{1}$ and $M_{2}\tau_{2}$ data symbols to $R_{1}$ from $T_{2}$ in $\tau_{2}$ time slots. With CSI matrices of Phase I, the relay re-constructs the Phase I output at receivers, i.e., $y_{1}^{P\text{-}I}$ and $y_{2}^{P\text{-}I}$. At each time slot, the data symbols for $R_{1}$ are sent from two transmitters, and simultaneously, the Phase I output at $R_{1}$ is sent from the relay. Denote the data symbols for $R_{1}$ sent from $T_{1}$ and $T_{2}$ as $s_{1,1}\in C^{M_{1}\tau_{2}}$ and $s_{2,1}\in C^{M_{2}\tau_{2}}$, respectively. Therefore, the transmit signals of Phase II at two transmitters are expressed as follows:

(10) $$x_{i}^{P\text{-}\mathrm{II}}=s_{i,1},\;i=1,2.$$

The transmit signals of Phase-II at the relay are designed as follows:

(11) $$x_{r}^{P\text{-}\mathrm{II}}=\Phi_{1}y_{1}^{P\text{-}I}.$$

The received signals of Phase-II at two receivers are written by the following:

(12) $$y_{j}^{P\text{-}\mathrm{II}}=H_{1,j}^{P\text{-}\mathrm{II}}s_{1,1}+H_{2,j}^{P\text{-}\mathrm{II}}s_{2,1}+H_{r,j}^{P\text{-}\mathrm{II}}\Phi_{1}y_{1}^{P\text{-}I},\;j=1,2.$$

*Phase III (joint data transmission for $R_{2}$ by two transmitters and Phase I output transmission by the relay)*: This phase aims at sending $M_{1}\tau_{3}$ data symbols to $R_{2}$ from $T_{1}$ and $M_{2}\tau_{3}$ data symbols to $R_{2}$ from $T_{2}$ in $\tau_{3}$ time slots. At each time slot, the data symbols for $R_{2}$ are sent from two transmitters, and simultaneously, the Phase I output at $R_{2}$ is sent from the relay. Denote the data symbols for $R_{2}$ sent from $T_{1}$ and $T_{2}$ as $s_{1,2}\in C^{M_{1}\tau_{3}}$ and $s_{2,2}\in C^{M_{2}\tau_{3}}$, respectively. Therefore, the transmit signals of Phase III at two transmitters are expressed as follows:

(13) $$x_{i}^{P\text{-}\mathrm{III}}=s_{i,2},\;i=1,2.$$

The transmit signals of Phase III at the relay are designed as follows:

(14) $$x_{r}^{P\text{-}\mathrm{III}}=\Phi_{2}y_{2}^{P\text{-}I}.$$

The received signals of Phase III at two receivers are written by the following:

(15) $$y_{j}^{P\text{-}\mathrm{III}}=H_{1,j}^{P\text{-}\mathrm{III}}s_{1,2}+H_{2,j}^{P\text{-}\mathrm{III}}s_{2,2}+H_{r,j}^{P\text{-}\mathrm{III}}\Phi_{2}y_{2}^{P\text{-}I},\;j=1,2.$$

*Security analysis*: Firstly, we need to verify the zero information leakage at $R_{1}$, when SNR goes to infinity. With $y_{1}≜\left[y_{1}^{P\text{-}I};y_{1}^{P\text{-}\mathrm{II}};y_{1}^{P\text{-}\mathrm{III}}\right]$, the information leakage is calculated as follows:

(16) $$\begin{array}{cc} \; & I(s_{1,2},s_{2,2};y_{1}|H^{n},s_{1,1},s_{2,1})\\ \; & \overset{\left(a\right)}{\leq }I\left(s_{1,2},s_{2,2},u;y_{1}|H^{n},s_{1,1},s_{2,1}\right)-I\left(u;y_{1}|H^{n},s_{1,1},s_{2,1},s_{1,2},s_{2,2}\right)\\ \; & \overset{\left(b\right)}{\leq }I\left(H_{r,1}^{P\text{-}I}u,H_{1,1}^{P\text{-}\mathrm{III}}s_{1,2}+H_{2,1}^{P\text{-}\mathrm{III}}s_{2,2}+H_{r,1}^{P\text{-}\mathrm{III}}\Phi_{2}y_{2}^{P\text{-}I};y_{1}|H^{n},s_{1,1},s_{2,1}\right)-I\left(u;y_{1}|H^{n},s_{1,1},s_{2,1},s_{1,2},s_{2,2}\right)\\ \; & \underset{\eta \to 1}{\overset{\left(c\right)}{=}}\mathrm{RK}\underset{A_{1}}{\underset{︸}{\left[\begin{array}{cc} I_{N_{1}\tau_{1}} & 0\\ H_{r,1}^{P\text{-}\mathrm{II}}\Phi_{1} & 0\\ 0 & I_{N_{1}\tau_{3}} \end{array}\right]}}\log\eta -\mathrm{RK}\underset{A_{2}}{\underset{︸}{\left[\begin{array}{c} H_{r,1}^{P\text{-}I}\\ H_{r,1}^{P\text{-}\mathrm{II}}\Phi_{1}H_{r,1}^{P\text{-}I}\\ H_{r,1}^{P\text{-}\mathrm{III}}\Phi_{2}H_{r,2}^{P\text{-}I} \end{array}\right]}}\log\eta \\ \; & \overset{\left(d\right)}{=}N_{1}\left(\tau_{1}+\tau_{3}\right)\log\eta -\min\left\{N_{1}\tau_{1}+\min\left\{R,N_{1}\right\}\tau_{3},\left(N_{1}+N_{2}\right)\tau_{1},R\tau_{1}\right\}\log\eta , \end{array}$$

where the reason for each step is given as follows:

(a) Chain rule of mutual information.
(b) Applying the data processing inequality for the Markov chain $\left(s_{1,2},s_{2,2},u\right)\to (H_{r,1}^{P\text{-}I}u$, $H_{1,1}^{P\text{-}\mathrm{III}}s_{1,2}+H_{2,1}^{P\text{-}\mathrm{III}}s_{2,2}+H_{r,1}^{P\text{-}\mathrm{III}}\Phi_{2}y_{2}^{P\text{-}I})\to y_{1}$.
(c) When the input is the circularly symmetric complex Gaussian, according to [30], rewrite the mutual information into $\log|I+\eta A_{1}A_{1}^{H}|-\log|I+\eta A_{2}A_{2}^{H}|$, and using ([10], Lemma 2), when SNR goes to infinity.
(d) The rank of matrix $A_{1}$ is deduced by Gaussian elimination. The rank of matrix $A_{2}$ is derived in Appendix A.

We shall ensure $I\left(s_{1,2},s_{2,2};y_{1}|H^{n},s_{1,1},s_{2,1}\right)=o\left(\log\eta \right)$, or equivalently, (16) is zero. If $N_{1}<R$, $N_{1}\left(\tau_{1}+\tau_{3}\right)=N_{1}\tau_{1}+\min\left\{R,N_{1}\right\}\tau_{3}$. If $N_{1}+N_{2}\leq R$, we have $N_{1}\left(\tau_{1}+\tau_{3}\right)=\left(N_{1}+N_{2}\right)\tau_{1}$. Otherwise, if $N_{1}<R<N_{1}+N_{2}$, we have $N_{1}\left(\tau_{1}+\tau_{3}\right)=R\tau_{1}$. Those two equalities can be simplified to the following:

(17) $$\frac{\tau_{1}}{\tau_{3}}=\frac{N_{1}}{\min\{N_{2},R-N_{1}\}}.$$

Otherwise, if $R\leq N_{1}$, we cannot guarantee zero information leakage from transmitted data symbols $s_{2,1},s_{2,1}$ to the undesired receiver $R_{2}$, which implies $\tau_{3}=0$.

Secondly, we only need to verify the zero information leakage at the $R_{2}$, when SNR goes to infinity. With $y_{2}≜\left[y_{2}^{P\text{-}I};y_{2}^{P\text{-}\mathrm{II}};y_{2}^{P\text{-}\mathrm{III}}\right]$, the information leakage is calculated as follows:

(18) $$\begin{array}{cc} \; & I(s_{1,1},s_{2,1};y_{2}|H^{n},s_{1,2},s_{2,2})\\ \; & \overset{\left(a\right)}{\leq }I\left(s_{1,1},s_{2,1},u;y_{2}|H^{n},s_{1,2},s_{2,2}\right)-I\left(u;y_{2}|H^{n},s_{1,1},s_{2,1},s_{1,2},s_{2,2}\right)\\ \; & \overset{\left(b\right)}{\leq }I\left(H_{r,2}^{P\text{-}I}u,H_{1,2}^{P\text{-}\mathrm{II}}s_{1,1}+H_{2,2}^{P\text{-}\mathrm{II}}s_{2,1}+H_{r,2}^{P\text{-}\mathrm{II}}\Phi_{1}y_{1}^{P\text{-}I};y_{2}|H^{n},s_{1,2},s_{2,2}\right)-I\left(u;y_{2}|H^{n},s_{1,1},s_{2,1},s_{1,2},s_{2,2}\right)\\ \; & \underset{\eta \to 1}{\overset{\left(c\right)}{=}}\mathrm{RK}\underset{B_{1}}{\underset{︸}{\left[\begin{array}{cc} I_{N_{2}\tau_{1}} & 0\\ 0 & I_{N_{2}\tau_{2}}\\ H_{r,2}^{P\text{-}\mathrm{III}}\Phi_{2} & 0 \end{array}\right]}}\log\eta -\mathrm{RK}\underset{B_{2}}{\underset{︸}{\left[\begin{array}{c} H_{r,2}^{P\text{-}I}\\ H_{r,2}^{P\text{-}\mathrm{II}}\Phi_{1}H_{r,1}^{P\text{-}I}\\ H_{r,2}^{P\text{-}\mathrm{III}}\Phi_{2}H_{r,2}^{P\text{-}I} \end{array}\right]}}\log\eta \\ \; & \overset{\left(d\right)}{=}N_{2}\left(\tau_{1}+\tau_{2}\right)\log\eta -\min\left\{N_{2}\tau_{1}+\min\left\{R,N_{2}\right\}\tau_{2},\left(N_{1}+N_{2}\right)\tau_{1},R\tau_{1}\right\}\log\eta , \end{array}$$

where the reason for each step is given as follows:

(a) Chain rule of mutual information.
(b) Applying the data processing inequality for the Markov chain $\left(s_{1,1},s_{2,1},u\right)\to (H_{r,2}^{P\text{-}I}u$, $H_{1,2}^{P\text{-}\mathrm{II}}s_{1,1}+H_{2,2}^{P\text{-}\mathrm{II}}s_{2,1}+H_{r,2}^{P\text{-}\mathrm{II}}\Phi_{1}y_{1}^{P\text{-}I})\to y_{2}$.
(c) When the input is the circularly symmetric complex Gaussian, according to [30], and rewrite the mutual information into $\log|I+\eta B_{1}B_{1}^{H}|-\log|I+\eta B_{2}B_{2}^{H}|$, and using ([10], Lemma 2), when SNR goes to infinity.
(d) The rank of matrix $B_{1}$ is deduced by Gaussian elimination. The rank of matrix $B_{2}$ is derived in Appendix A.

We shall ensure $I\left(s_{1,1},s_{2,1};y_{2}|H^{n},s_{1,2},s_{2,2}\right)=o\left(\log\eta \right)$, or equivalently, (18) is zero. If $N_{2}<R$, $N_{2}\left(\tau_{1}+\tau_{2}\right)\leq N_{2}\tau_{1}+\min\left\{R,N_{2}\right\}\tau_{2}$. If $N_{1}+N_{2}\leq R$, we have $N_{2}\left(\tau_{1}+\tau_{2}\right)=\left(N_{1}+N_{2}\right)\tau_{1}$. Otherwise, if $N_{2}<R<N_{1}+N_{2}$, we have $N_{2}\left(\tau_{1}+\tau_{2}\right)=R\tau_{1}$. Those two inequalities can be simplified to the following:

(19) $$\frac{\tau_{1}}{\tau_{2}}=\frac{N_{2}}{\min\{N_{1},R-N_{2}\}}.$$

If $R\leq N_{2}$, we cannot guarantee zero information leakage from transmitted data symbols $s_{1,1},s_{1,2}$ to the undesired receiver $R_{2}$, which implies $\tau_{2}=0$.

*Decoding analysis*: Firstly, we need to verify the decoding of transmitted data symbols $s_{1,1}\in C^{M_{1}\tau_{2}},s_{2,1}\in C^{M_{2}\tau_{2}}$ at $R_{1}$, which is enabled by the following cancellation:

(20) $$y_{1}^{P\text{-}\mathrm{II}}-H_{r,1}^{P\text{-}\mathrm{II}}\Phi_{1}y_{1}^{P\text{-}I}=H_{1,1}^{P\text{-}\mathrm{II}}s_{1,1}+H_{2,1}^{P\text{-}\mathrm{II}}s_{2,1},$$

At each time slot, there are $N_{1}$ observations at $R_{1}$. Thus, we are able to decode $M_{1}$ data symbols from $T_{1}$ and $M_{2}$ data symbols from $T_{2}$, due to the setting, i.e., $M_{1}+M_{2}\leq N_{1}$. Therefore, $R_{1}$ can decode $\left(M_{1}+M_{2}\right)\tau_{2}$ data symbols.

Secondly, we need to verify the decoding of transmitted data symbols $s_{1,2}\in C^{M_{1}\tau_{3}},s_{2,2}\in C^{M_{2}\tau_{3}}$ at $R_{2}$, which is enabled by the following cancellation:

(21) $$y_{2}^{P\text{-}\mathrm{III}}-H_{r,2}^{P\text{-}\mathrm{III}}\Phi_{2}y_{2}^{P\text{-}I}=H_{1,2}^{P\text{-}\mathrm{III}}s_{1,2}+H_{2,2}^{P\text{-}\mathrm{III}}s_{2,2},$$

At each time slot, there are $N_{2}$ observations at $R_{2}$. Thus, we are able to decode $M_{1}$ data symbols from $T_{1}$ and $M_{2}$ data symbols from $T_{2}$, due to the setting, i.e., $M_{1}+M_{2}\leq N_{1}\leq N_{2}$. Therefore, $R_{2}$ can decode $\left(M_{1}+M_{2}\right)\tau_{3}$ data symbols.

*Achievable sum-SDoF analysis*: As shown in the decoding analysis, two receivers can decode a total of $\left(M_{1}+M_{2}\right)\left(\tau_{2}+\tau_{3}\right)$ data symbols over $\tau_{1}+\tau_{2}+\tau_{3}$ time slots. This implies that the achievable sum-SDoF can be expressed as $\left(M_{1}+M_{2}\right)\left(\tau_{2}+\tau_{3}\right)/\left(\tau_{1}+\tau_{2}+\tau_{3}\right)$. According to the security analysis, the achievable sum-SDoF of proposed relay-aided jamming scheme is calculated through the following:

- If $N_{2}<R$, we substitute (17) and (19) into $\left(M_{1}+M_{2}\right)\left(\tau_{2}+\tau_{3}\right)/\left(\tau_{1}+\tau_{2}+\tau_{3}\right)$ to derive the exact achievable sum-SDoF.
- If $N_{1}<R\leq N_{2}$, we substitute $\tau_{2}=0$ and (17) into $\left(M_{1}+M_{2}\right)\left(\tau_{2}+\tau_{3}\right)/\left(\tau_{1}+\tau_{2}+\tau_{3}\right)$ to derive the exact achievable sum-SDoF.
- If $R\leq N_{1}$, we cannot ensure zero information leakage; hence, achievable sum-SDoF is zero.

The achievable sum-SDoF of the proposed relay-aided jamming scheme is given in (4) for $M_{1}+M_{2}\leq N_{1}\;\;\&\;\;N_{2}<R$ Case, $M_{1}+M_{2}\leq N_{1}\;\;\&\;\;N_{1}<R\leq N_{2}$ Case, and $R\leq N_{1}$ Case.

### 4.2. $N_{1}<M_{1}+M_{2}\leq N_{2}$ Case: The Relay-Aided Jamming and One-Receiver IA Scheme

**Figure 8:**
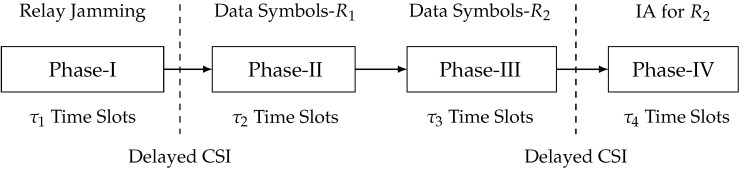
Flowchart of the relay-aided jamming and one-receiver IA scheme.

When $N_{1}<M_{1}+M_{2}\leq N_{2}$, then $R_{1}$ can immediately decode the transmitted data symbols, while $R_{2}$ cannot. Therefore, the technique of IA can be adopted to enable the decoding of transmitted data symbols for $R_{2}$, where the interference at $R_{1}$ is re-transmitted to provide lacking equations for decoding. To this end, we propose a relay-aided jamming and the one-receiver IA scheme with four phases, where the relay cooperates with two transmitters for both artificial noise transmission and IA. In Phase I, the relay sends artificial noise symbols. In Phase II, two transmitters send data symbols for $R_{1}$; meanwhile, the output signal of Phase I at $R_{1}$ is reconstructed and re-transmitted by relay. In Phase III, two transmitters send data symbols for $R_{2}$; meanwhile, the output signal of Phase I at $R_{2}$ is reconstructed and re-transmitted by relay. In Phase IV, the interference at $R_{2}$ is re-transmitted to provide lacking equations for $R_{1}$. The flowchart of this scheme is illustrated in Figure 8. Firstly, we define the holistic CSI matrices for this scheme as follows:

$$\begin{array}{ccc} & & H_{i,j}^{P\text{-}I}≜\mathrm{BD}\left\{H_{i,j}\left[1\right],\cdots ,H_{i,j}\left[\tau_{1}\right]\right\},\\ & & H_{i,j}^{P\text{-}\mathrm{II}}≜\mathrm{BD}\left\{H_{i,j}\left[\tau_{1}+1\right],\cdots ,H_{i,j}\left[\tau_{1}+\tau_{2}\right]\right\},\\ & & H_{i,j}^{P\text{-}\mathrm{III}}≜\mathrm{BD}\left\{H_{i,j}\left[\tau_{1}+\tau_{2}+1\right],\cdots ,H_{i,j}\left[\tau_{1}+\tau_{2}+\tau_{3}\right]\right\},\\ & & H_{i,j}^{P\text{-}\mathrm{IV}}≜\mathrm{BD}\left\{H_{i,j}\left[\tau_{1}+\tau_{2}+\tau_{3}+1\right],\cdots ,H_{i,j}\left[\tau_{1}+\tau_{2}+\tau_{3}+\tau_{4}\right]\right\}, \end{array}$$

where i,j=1,2,r, and the value of $\tau_{1}$, $\tau_{2}$, and $\tau_{3}$ is determined based on security analysis of the scheme. Moreover, we introduce the pre-stored full-rank matrices $\Phi_{1}\in C^{R\tau_{2}\times N_{1}\tau_{1}}$, $\Phi_{2}\in C^{R\tau_{3}\times N_{2}\tau_{1}}$, and $\Pi_{1}\in C^{\min\left\{N_{1},R\right\}\tau_{4}\times N_{2}\tau_{2}}$, whose items follow complex Gaussian CN(0,1). Next, the proposed three-phase transmission scheme and related analysis are elaborated.

*Phase I (jamming by the relay)*: This phase aims at sending $R\tau_{1}$ artificial noise symbols from the relay in $\tau_{1}$ time slots by *R* antennas. Denote the artificial noise vector sent by *R* antennas in $\tau_{1}$ time slots as $u\in C^{R\tau_{1}}$. The received signals of Phase I at two receivers are expressed as follows:

(22) $$y_{j}^{P\text{-}I}=H_{r,j}^{P\text{-}I}u,\;j=1,2.$$

The CSI matrices of Phase I return to the relay at the end of Phase I.

*Phase II (joint data transmission for $R_{1}$ by two transmitters and Phase I output transmission by the relay)*: We aim at sending $M_{1}\tau_{2}$ data symbols to $R_{1}$ from $T_{1}$ and $\min\left\{M_{2},R-M_{1}\right\}\tau_{2}$ data symbols to $R_{1}$ from $T_{2}$ in $\tau_{2}$ time slots. With CSI matrices of Phase I, the relay re-constructs the Phase I output at the receivers, i.e., $y_{1}^{P\text{-}I}$ and $y_{2}^{P\text{-}I}$. At each time slot, the data symbols for $R_{1}$ are sent from two transmitters, and simultaneously, the Phase I output at $R_{1}$ is sent from the relay. Denote the data symbols for $R_{1}$ sent from $T_{1}$ and $T_{2}$ as $s_{1,1}\in C^{M_{1}\tau_{2}}$ and $s_{2,1}\in C^{\min\left\{M_{2},R-M_{1}\right\}\tau_{2}}$, respectively. Therefore, the transmit signals of Phase-II at two transmitters are expressed as follows:

(23) $$x_{i}^{P\text{-}\mathrm{II}}=s_{i,1},\;i=1,2.$$

The transmit signals of Phase II at the relay are designed as follows:

(24) $$x_{r}^{P\text{-}\mathrm{II}}=\Phi_{1}y_{1}^{P\text{-}I}.$$

The received signals of Phase II at two receivers are written as follows:

(25) $$y_{j}^{P\text{-}\mathrm{II}}=H_{1,j}^{P\text{-}\mathrm{II}}s_{1,1}+H_{2,j}^{P\text{-}\mathrm{II}}s_{2,1}+H_{r,j}^{P\text{-}\mathrm{II}}\Phi_{1}y_{1}^{P\text{-}I},\;j=1,2.$$

After the successful self-interference cancellation (for the successful interference cancellation, the residual self-interference can achieve noise floor; therefore, the residual self-interference does not affect the SDoF analysis) at the relay, the received signals of Phase II at the relay are expressed as follows:

(26) $$y_{r}^{P\text{-}\mathrm{II}}=H_{1,r}^{P\text{-}\mathrm{II}}s_{1,2}+H_{2,r}^{P\text{-}\mathrm{II}}s_{2,2}.$$

Since there are *R* observations at the relay, the relay can immediately decode all transmitted $\min\{M_{1}+M_{2},R\}$ data symbols, given by $s_{1,1}$ and $s_{2,1}$. The CSI matrices of Phase II return to the relay at the end of Phase II.

*Phase III (joint data transmission for $R_{2}$ by two transmitters and Phase I output transmission by the relay)*: This phase aims at sending $M_{1}\tau_{3}$ data symbols to $R_{2}$ from $T_{1}$ and $M_{2}\tau_{3}$ data symbols to $R_{2}$ from $T_{2}$ in $\tau_{3}$ time slots. At each time slot, the data symbols for $R_{2}$ are sent from two transmitters, and simultaneously, the Phase I output at $R_{2}$ is sent from the relay. Denote the data symbols for $R_{1}$ sent from $T_{1}$ and $T_{2}$ as $s_{1,2}\in C^{M_{1}\tau_{3}}$ and $s_{2,2}\in C^{M_{2}\tau_{3}}$, respectively. Therefore, the transmit signals of Phase III at the two transmitters are expressed as follows:

(27) $$x_{i}^{P\text{-}\mathrm{III}}=s_{i,2},\;i=1,2.$$

The transmit signals of Phase III at the relay are designed as follows:

(28) $$x_{r}^{P\text{-}\mathrm{III}}=\Phi_{2}y_{2}^{P\text{-}I}.$$

The received signals of Phase III at two receivers are written as follows:

(29) $$y_{j}^{P\text{-}\mathrm{III}}=H_{1,j}^{P\text{-}\mathrm{III}}s_{1,2}+H_{2,j}^{P\text{-}\mathrm{III}}s_{2,2}+H_{r,j}^{P\text{-}\mathrm{III}}\Phi_{2}y_{2}^{P\text{-}I},\;j=1,2.$$

The CSI matrices of Phase III return to the relay at the end of Phase III.

*Phase IV (one-receiver IA by the relay)*: In order to provide lacking equations for decoding, we aim at sending $\min\left\{N_{1},R\right\}\tau_{3}$ combinations of interference at Phase II in $\tau_{4}$ time slots. With CSI matrices of Phase I and Phase II, $s_{1,1}$ and $s_{2,1}$, the relay re-constructs the interference at Phase II, i.e., $y_{2}^{P\text{-}\mathrm{II}}$. The transmit signals of Phase IV at the relay are designed as follows:

(30) $$x_{r}^{P\text{-}\mathrm{IV}}=\Pi_{1}y_{2}^{P\text{-}\mathrm{II}}.$$

At the same time, two transmitters keep silent. The received signals of Phase IV at two receivers are written as follows:

(31) $$y_{j}^{P\text{-}\mathrm{IV}}=H_{r,j}^{P\text{-}\mathrm{IV}}\Pi_{1}y_{2}^{P\text{-}\mathrm{II}},\;j=1,2.$$

*Security Analysis*: As the received signals in Phase IV can be constructed by the received signals in Phase II and CSI matrices, the security analysis is similar to that in the relay-aided jamming scheme. Therefore, it can be checked that to ensure zero information leakage at $R_{1}$ and $R_{2}$, when SNR goes to infinity, phase duration should follow (17) and (19), respectively.

*Decoding analysis*: Firstly, we need to verify the decoding of transmitted data symbols $s_{1,1}$ and $s_{2,1}$ at $R_{1}$. This is enabled by the following cancellation:

(32) $$\left[\begin{array}{c} y_{1}^{P\text{-}\mathrm{II}}-H_{r,1}^{P\text{-}\mathrm{II}}\Phi_{1}y_{1}^{P\text{-}I}\\ y_{1}^{P\text{-}\mathrm{II}}-H_{r,1}^{P\text{-}\mathrm{IV}}\Pi_{1}H_{r,2}^{P\text{-}\mathrm{II}}\Phi_{1}y_{1}^{P\text{-}I} \end{array}\right]=\underset{H_{1}}{\underset{︸}{\left[\begin{array}{cc} H_{1,1}^{P\text{-}\mathrm{II}} & H_{2,1}^{P\text{-}\mathrm{II}}\\ H_{r,1}^{P\text{-}\mathrm{IV}}\Pi_{1}H_{1,2}^{P\text{-}\mathrm{II}} & H_{r,1}^{P\text{-}\mathrm{IV}}\Pi_{1}H_{2,2}^{P\text{-}\mathrm{II}} \end{array}\right]}}\left[\begin{array}{c} s_{1,1}\\ s_{2,1} \end{array}\right],$$

where the rank of matrix $H_{1}$ is $\min\{N_{1}\tau_{2}+\min\left\{N_{1}\tau_{4},R\tau_{4}\right\},\left(M_{1}+M_{2}\right)\tau_{2},R\tau_{2}\}$. The reason is given in Appendix B. Since $\min\left\{M_{1}+M_{2},R\right\}\tau_{2}$ data symbols for $R_{1}$ are transmitted, to ensure the decoding, we shall follow that

(33) $$\min\left\{M_{1}+M_{2},R\right\}\tau_{2}\leq N_{1}\tau_{2}+\min\left\{N_{1}\tau_{4},R\tau_{4}\right\},$$

which simplifies to the following:

(34) $$\frac{\tau_{2}}{\tau_{4}}\leq \frac{\min\{N_{1},R\}}{\min\{M_{1}+M_{2}-N_{1},R-N_{1}\}}.$$

Then, multiplying (34) by (19), we have the following:

(35) $$\frac{\tau_{1}}{\tau_{4}}\leq \frac{\min\left\{N_{1},R\right\}N_{2}}{\min\left\{M_{1}+M_{2}-N_{1},R-N_{1}\right\}\min\left\{N_{1},R-N_{2}\right\}}.$$

Secondly, we need to verify the decoding of transmitted data symbols $s_{1,2}$ and $s_{2,2}$ at the receiver $R_{2}$, which is enabled by the following cancellation:

(36) $$y_{2}^{P\text{-}\mathrm{III}}-H_{r,2}^{P\text{-}\mathrm{III}}\Phi_{2}y_{2}^{P\text{-}I}=H_{1,2}^{P\text{-}\mathrm{III}}s_{1,2}+H_{2,2}^{P\text{-}\mathrm{III}}s_{2,2},$$

At each time slot, there are $N_{2}$ observations at $R_{2}$. Thus, we are able to decode $M_{1}$ data symbols from $T_{1}$ and $M_{2}$ data symbols from $T_{2}$, due to the setting, i.e, $M_{1}+M_{2}\leq N_{2}$. Therefore, $R_{2}$ can decode $\left(M_{1}+M_{2}\right)\tau_{3}$ data symbols.

*Achievable sum-SDoF analysis*: As shown in the decoding analysis, two receivers can decode a total of $\min\left\{M_{1}+M_{2},R\right\}\tau_{2}+\left(M_{1}+M_{2}\right)\tau_{3}$ data symbols over $\tau_{1}+\tau_{2}+\tau_{3}+\tau_{4}$ time slots. This implies that the achievable sum-SDoF can be expressed as $\left(\min\left\{M_{1}+M_{2},R\right\}\tau_{2}+\left(M_{1}+M_{2}\right)\tau_{3}\right)/\left(\tau_{1}+\tau_{2}+\tau_{3}+\tau_{4}\right)$. According to the security and decoding analysis, the achievable sum-SDoF of proposed relay-aided jamming and one-receiver IA scheme is calculated through the following:

- If $N_{2}<R$, we substitute (17), (19), and (35) with equality into $\left(\min\left\{M_{1}+M_{2},R\right\}\tau_{2}+\left(M_{1}+M_{2}\right)\tau_{3}\right)/\left(\tau_{1}+\tau_{2}+\tau_{3}+\tau_{4}\right)$ to derive the exact achievable sum-SDoF.
- If $N_{1}<R\leq N_{2}$, we substitute (17), $\tau_{2}=0$, and $\tau_{4}=0$ into $\left(\min\left\{M_{1}+M_{2},R\right\}\tau_{2}+\left(M_{1}+M_{2}\right)\tau_{3}\right)/\left(\tau_{1}+\tau_{2}+\tau_{3}+\tau_{4}\right)$ to derive the exact achievable sum-SDoF.
- If $R\leq N_{1}$, we cannot ensure zero information leakage, and hence, the achievable sum-SDoF is zero.

The achievable sum-SDoF of the proposed relay-aided jamming and one-receiver IA scheme is given in (4) for $N_{1}<M_{1}+M_{2}\leq N_{2}\;\;\&\;\;N_{2}<R$ Case, $M_{1}+M_{2}\leq N_{2}\;\;\&\;\;N_{1}<R\leq N_{2}$ Case, and $R\leq N_{1}$ Case.

### 4.3. $N_{2}<M_{1}+M_{2}$ Case: The Relay-Aided Jamming and Two-Receiver IA Scheme

**Figure 9:**
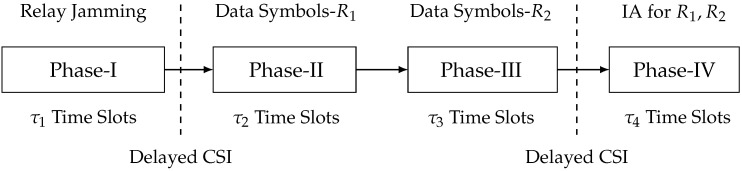
Flowchart of the relay-aided jamming and two-receiver IA scheme.

When $N_{2}<M_{1}+M_{2}$, both two receivers, i.e., $R_{1}$ and $R_{2}$, are unable to immediately decode the transmitted data symbols. Therefore, the technique of IA can be adopted to enable the decoding of transmitted data symbols for both two receivers, where the interference at both two receivers is re-transmitted to provide lacking equations for decoding. To this end, we propose a relay-aided jamming and two-receiver IA scheme with four phases, where the relay cooperates with two transmitters for both artificial noise transmission and IA. In Phase I, the relay sends artificial noise symbols. In Phase II, two transmitters send data symbols for $R_{1}$; meanwhile, the output signal of Phase I at $R_{1}$ is reconstructed and re-transmitted by relay. In Phase III, two transmitters send data symbols for $R_{2}$; meanwhile the output signal of Phase I at $R_{2}$ is reconstructed and re-transmitted by relay. In Phase IV, the interference at $R_{2}$ is re-transmitted to provide lacking equations for $R_{1}$; meanwhile, the interference at $R_{1}$ is re-transmitted to provide lacking equations for $R_{2}$. The flowchart of this scheme is illustrated in Figure 9. Firstly, we define the holistic CSI matrices as follows:

$$\begin{array}{ccc} & & H_{i,j}^{P\text{-}I}≜\mathrm{BD}\left\{H_{i,j}\left[1\right],\cdots ,H_{i,j}\left[\tau_{1}\right]\right\},\\ & & H_{i,j}^{P\text{-}\mathrm{II}}≜\mathrm{BD}\left\{H_{i,j}\left[\tau_{1}+1\right],\cdots ,H_{i,j}\left[\tau_{1}+\tau_{2}\right]\right\},\\ & & H_{i,j}^{P\text{-}\mathrm{III}}≜\mathrm{BD}\left\{H_{i,j}\left[\tau_{1}+\tau_{2}+1\right],\cdots ,H_{i,j}\left[\tau_{1}+\tau_{2}+\tau_{3}\right]\right\},\\ & & H_{i,j}^{P\text{-}\mathrm{IV}}≜\mathrm{BD}\left\{H_{i,j}\left[\tau_{1}+\tau_{2}+\tau_{3}+1\right],\cdots ,H_{i,j}\left[\tau_{1}+\tau_{2}+\tau_{3}+\tau_{4}\right]\right\}, \end{array}$$

where i,j=1,2,r, and the values of $\tau_{1}$, $\tau_{2}$, $\tau_{3}$, and $\tau_{4}$ are determined based on security and decoding analysis of the scheme. Moreover, we introduce the pre-stored full-rank matrices $\Phi_{1}\in C^{R\tau_{2}\times N_{1}\tau_{1}}$, $\Phi_{1}\in C^{R\tau_{3}\times N_{2}\tau_{1}}$, $\Pi_{1}\in C^{\min\left\{N_{1},R\right\}\tau_{4}\times N_{2}\tau_{2}}$, and $\Pi_{2}\in C^{\min\left\{N_{1},R\right\}\tau_{4}\times N_{1}\tau_{3}}$, whose items follow complex Gaussian CN(0,1). Next, the proposed four-phase transmission scheme and related analysis are elaborated.

*Phase I (jamming by the relay)*: This phase aims at sending $R\tau_{1}$ artificial noise symbols from the relay by *R* antennas in $\tau_{1}$ time slots. Denote the artificial noise vector sent by *R* antennas in $\tau_{1}$ time slots as $u\in C^{R\tau_{1}}$. The received signals of Phase I at two receivers are expressed as follows:

(37) $$y_{j}^{P\text{-}I}=H_{r,j}^{P\text{-}I}u,\;j=1,2.$$

The CSI matrices of Phase I return to the relay at the end of Phase I.

*Phase II (joint data transmission for $R_{1}$ by two transmitters and Phase I output transmission by the relay)*: We aim at sending $M_{1}\tau_{2}$ data symbols to $R_{1}$ from $T_{1}$ and $\min\left\{M_{2},R-M_{1},N_{1}+N_{2}-M_{1}\right\}\tau_{2}$ data symbols to $R_{1}$ from $T_{2}$ in $\tau_{2}$ time slots. With CSI matrices of Phase I, the relay re-constructs the Phase I output at receivers, i.e., $y_{1}^{P\text{-}I}$ and $y_{2}^{P\text{-}I}$. At each time slot, the data symbols for $R_{1}$ are sent from two transmitters, and simultaneously, the Phase I output at $R_{1}$ is sent from the relay. Denote the data symbols for $R_{1}$ sent from $T_{1}$ and $T_{2}$ as $s_{1,1}\in C^{M_{1}\tau_{2}}$ and $s_{2,1}\in C^{\min\left\{M_{2},R-M_{1},N_{1}+N_{2}-M_{1}\right\}\tau_{2}}$, respectively. Therefore, the transmit signals of Phase II at two transmitters are expressed as follows:

(38) $$x_{i}^{P\text{-}\mathrm{II}}=s_{i,1},\;i=1,2.$$

The transmit signals of Phase II at the relay are designed as follows:

(39) $$x_{r}^{P\text{-}\mathrm{II}}=\Phi_{1}y_{1}^{P\text{-}I}.$$

The received signals of Phase II at two receivers are written as follows:

(40) $$y_{j}^{P\text{-}\mathrm{II}}=H_{1,j}^{P\text{-}\mathrm{II}}s_{1,1}+H_{2,j}^{P\text{-}\mathrm{II}}s_{2,1}+H_{r,j}^{P\text{-}\mathrm{II}}\Phi_{1}y_{1}^{P\text{-}I},\;j=1,2.$$

After the successful self-interference cancellation at the relay, the received signals of Phase II at the relay are expressed as follows:

(41) $$y_{r}^{P\text{-}\mathrm{II}}=H_{1,r}^{P\text{-}\mathrm{II}}s_{1,1}+H_{2,r}^{P\text{-}\mathrm{II}}s_{2,1}.$$

Since there are *R* observations at the relay, the relay can immediately decode all transmitted $\min\{M_{1}+M_{2},R,N_{1}+N_{2}\}$ data symbols, given by $s_{1,1}$ and $s_{2,1}$. The CSI matrices of Phase II return to the relay at the end of Phase II.

*Phase III (joint data transmission for $R_{2}$ by two transmitters and Phase I output transmission by the relay)*: We aim at sending $M_{1}\tau_{2}$ data symbols to $R_{2}$ from $T_{1}$ and $\min\left\{M_{2},R-M_{1},N_{1}+N_{2}-M_{1}\right\}\tau_{2}$ data symbols to $R_{2}$ from $T_{2}$ in $\tau_{3}$ time slots. At each time slot, the data symbols for $R_{2}$ are sent from two transmitters, and simultaneously, the Phase I output at $R_{2}$ is sent from the relay. Denote the data symbols for $R_{2}$ sent from $T_{1}$ and $T_{2}$ as $s_{1,2}\in C^{M_{1}\tau_{2}}$ and $s_{2,2}\in C^{\min\left\{M_{2},R-M_{1},N_{1}+N_{2}-M_{1}\right\}\tau_{2}}$, respectively. Therefore, the transmit signals of Phase-III at two transmitters are expressed as follows:

(42) $$x_{i}^{P\text{-}\mathrm{III}}=s_{i,2},\;i=1,2.$$

The transmit signals of Phase III at the relay are designed as follows:

(43) $$x_{r}^{P\text{-}\mathrm{III}}=\Phi_{2}y_{2}^{P\text{-}I}.$$

The received signals of Phase II at two receivers are written as follows:

(44) $$y_{j}^{P\text{-}\mathrm{III}}=H_{1,j}^{P\text{-}\mathrm{III}}s_{1,2}+H_{2,j}^{P\text{-}\mathrm{III}}s_{2,2}+H_{r,j}^{P\text{-}\mathrm{III}}\Phi_{2}y_{2}^{P\text{-}I},\;j=1,2.$$

After the successful self-interference cancellation at the relay, the received signals of Phase II at the relay are expressed as follows:

(45) $$y_{r}^{P\text{-}\mathrm{III}}=H_{1,r}^{P\text{-}\mathrm{III}}s_{1,2}+H_{2,r}^{P\text{-}\mathrm{III}}s_{2,2}.$$

Since there are *R* observations at the relay, the relay can immediately decode all transmitted $\min\{M_{1}+M_{2},R,N_{1}+N_{2}\}$ data symbols, given by $s_{1,1}$ and $s_{2,1}$. The CSI matrices of Phase III return to the relay at the end of Phase III.

*Phase IV (two-receiver IA by the relay)*: In order to provide lacking equations for decoding, we aim at sending $\min\left\{N_{1},R\right\}\tau_{4}$ combinations of interference at Phase II and Phase III by $\min\{N_{1},R\}$ antennas in $\tau_{4}$ time slots after Phase III. With CSI matrices of Phase I, Phase II, and Phase III, the relay re-constructs the interference at Phase II and Phase III, i.e, $y_{2}^{P\text{-}\mathrm{II}}$ and $y_{1}^{P\text{-}\mathrm{III}}$. The transmit signals of Phase-IV at the relay are designed as follows:

(46) $$x_{r}^{P\text{-}\mathrm{IV}}=\Pi_{2}y_{1}^{P\text{-}\mathrm{III}}+\Pi_{1}y_{2}^{P\text{-}\mathrm{II}}.$$

At the same time, two transmitters keep silent. The received signals of Phase IV at two receivers are written by the following:

(47) $$y_{j}^{P\text{-}\mathrm{IV}}=H_{r,j}^{P\text{-}\mathrm{IV}}\Pi_{2}y_{1}^{P\text{-}\mathrm{III}}+H_{r,j}^{P\text{-}\mathrm{IV}}\Pi_{1}y_{2}^{P\text{-}\mathrm{II}},\;j=1,2.$$

*Security Analysis*: As the received signals in Phase IV can be constructed by the received signals in Phase II, Phase III, and CSI matrices, the security analysis is similar to that in the relay-aided jamming scheme. Therefore, it can be checked that to ensure zero information leakage at $R_{1}$ and $R_{2}$, when SNR goes to infinity, the phase duration should follow (17) and (19), respectively.

*Decoding analysis*: Firstly, we need to verify the decoding of transmitted data symbols $s_{1,1}$ and $s_{2,1}$ at the receiver $R_{1}$. This is enabled by the following cancellation:

(48) $$\begin{array}{ccc} & & \left[\begin{array}{c} y_{1}^{P\text{-}\mathrm{II}}-H_{r,1}^{P\text{-}\mathrm{II}}\Phi_{1}y_{1}^{P\text{-}I}\\ y_{1}^{P\text{-}\mathrm{IV}}-H_{r,1}^{P\text{-}\mathrm{IV}}\Pi_{2}y_{1}^{P\text{-}\mathrm{III}}-H_{r,1}^{P\text{-}\mathrm{IV}}\Pi_{1}H_{r,2}^{P\text{-}\mathrm{II}}\Phi_{1}y_{1}^{P\text{-}I} \end{array}\right]=\underset{H_{1}^{\prime }}{\underset{︸}{\left[\begin{array}{cc} H_{1,1}^{P\text{-}\mathrm{II}} & H_{2,1}^{P\text{-}\mathrm{II}}\\ H_{r,1}^{P\text{-}\mathrm{IV}}\Pi_{1}H_{1,2}^{P\text{-}\mathrm{II}} & H_{r,1}^{P\text{-}\mathrm{IV}}\Pi_{1}H_{2,2}^{P\text{-}\mathrm{II}} \end{array}\right]}}\left[\begin{array}{c} s_{1,1}\\ s_{2,1} \end{array}\right], \end{array}$$

where the rank of matrix $H_{1}^{\prime }$ is $\min\{N_{1}\tau_{2}+\min\left\{N_{1},R\right\}\tau_{4},\left(N_{1}+N_{2}\right)\tau_{2},\left(M_{1}+M_{2}\right)\tau_{2},R\tau_{2}\}$, and the reason is given in Appendix C. Since $\min\left\{M_{1}+M_{2},R,N_{1}+N_{2}\right\}\tau_{2}$ data symbols for $R_{1}$ are transmitted, to ensure the decoding, we shall follow that

(49) $$\min\left\{M_{1}+M_{2},R,N_{1}+N_{2}\right\}\tau_{2}\leq \min\left\{N_{1}\tau_{2}+\min\left\{N_{1},R\right\}\tau_{4},\left(N_{1}+N_{2}\right)\tau_{2}\right\}.$$

If $M_{1}+M_{2}\leq N_{1}+N_{2}$, (49) simplifies to the following:

(50) $$\frac{\tau_{2}}{\tau_{4}}\leq \frac{\min\{N_{1},R\}}{\min\{M_{1}+M_{2}-N_{1},R-N_{1}\}}.$$

Otherwise, if $N_{1}+N_{2}<M_{1}+M_{2}$, (49) simplifies to the following:

(51) $$\frac{\tau_{2}}{\tau_{4}}\leq \frac{\min\{N_{1},R\}}{\min\{N_{2},R-N_{1}\}}.$$

Combining (50) and (51), we have the following:

(52) $$\frac{\tau_{2}}{\tau_{4}}\leq \frac{\min\{N_{1},R\}}{\min\{M_{1}+M_{2}-N_{1},N_{2},R-N_{1}\}}.$$

Then, multiplying (52) by (19), we have the following:

(53) $$\frac{\tau_{1}}{\tau_{4}}\leq \frac{\min\left\{N_{1},R\right\}N_{2}}{\min\left\{M_{1}+M_{2}-N_{1},N_{2},R-N_{1}\right\}\min\left\{N_{1},R-N_{2}\right\}}.$$

Secondly, we need to verify the decoding of transmitted data symbols $s_{1,2}$ and $s_{2,2}$ at the receiver $R_{2}$, which is enabled by the following cancellation:

(54) $$\begin{array}{ccc} & & \left[\begin{array}{c} y_{2}^{P\text{-}\mathrm{III}}-H_{r,2}^{P\text{-}\mathrm{III}}\Phi_{2}y_{2}^{P\text{-}I}\\ y_{2}^{P\text{-}\mathrm{IV}}-H_{r,2}^{P\text{-}\mathrm{IV}}\Pi_{1}y_{2}^{P\text{-}\mathrm{II}}-H_{r,2}^{P\text{-}\mathrm{IV}}\Pi_{2}H_{r,1}^{P\text{-}\mathrm{III}}\Phi_{2}y_{2}^{P\text{-}I} \end{array}\right]=\underset{H_{2}^{\prime }}{\underset{︸}{\left[\begin{array}{cc} H_{1,2}^{P\text{-}\mathrm{III}} & H_{2,2}^{P\text{-}\mathrm{III}}\\ H_{r,2}^{P\text{-}\mathrm{IV}}\Pi_{2}H_{1,1}^{P\text{-}\mathrm{III}} & H_{r,2}^{P\text{-}\mathrm{IV}}\Pi_{2}H_{2,1}^{P\text{-}\mathrm{III}} \end{array}\right]}}\left[\begin{array}{c} s_{1,2}\\ s_{2,2} \end{array}\right], \end{array}$$

where the rank of matrix $H_{2}^{\prime }$ is $\min\{N_{2}\tau_{3}+\min\left\{N_{1},R\right\}\tau_{4},\left(N_{1}+N_{2}\right)\tau_{3},\left(M_{1}+M_{2}\right)\tau_{3},R\tau_{3}\}$, and the reason is given in Appendix C. Since $\min\left\{M_{1}+M_{2},R,N_{1}+N_{2}\right\}\tau_{3}$ data symbols for $R_{2}$ are transmitted, to ensure the decoding, we shall follow that

(55) $$\min\left\{M_{1}+M_{2},R,N_{1}+N_{2}\right\}\tau_{3}\leq \min\left\{N_{2}\tau_{3}+\min\left\{N_{1},R\right\}\tau_{4},\left(N_{1}+N_{2}\right)\tau_{3}\right\}.$$

If $M_{1}+M_{2}\leq N_{1}+N_{2}$, (55) simplifies to the following:

(56) $$\frac{\tau_{3}}{\tau_{4}}\leq \frac{\min\{N_{1},R\}}{\min\{M_{1}+M_{2}-N_{2},R-N_{2}\}}.$$

Otherwise, if $N_{1}+N_{2}<M_{1}+M_{2}$, (49) simplifies to the following:

(57) $$\frac{\tau_{3}}{\tau_{4}}\leq \frac{\min\{N_{1},R\}}{\min\{N_{1},R-N_{2}\}}.$$

Combining (56) and (57), we have the following:

(58) $$\frac{\tau_{3}}{\tau_{4}}\leq \frac{\min\{N_{1},R\}}{\min\{M_{1}+M_{2}-N_{2},N_{1},R-N_{2}\}}.$$

Then, multiplying (58) by (17), we have the following:

(59) $$\frac{\tau_{1}}{\tau_{4}}\leq \frac{\min\left\{N_{1},R\right\}N_{1}}{\min\left\{M_{1}+M_{2}-N_{2},N_{1},R-N_{2}\right\}\min\left\{N_{2},R-N_{1}\right\}}.$$

*Achievable sum-SDoF analysis*: As shown in the decoding analysis, two receivers can decode a total of $\min\left\{M_{1}+M_{2},R,N_{1}+N_{2}\right\}\left(\tau_{2}+\tau_{3}\right)$ data symbols over $\tau_{1}+\tau_{2}+\tau_{3}+\tau_{4}$ time slots. This implies that the achievable sum-SDoF can be expressed as $\min\left\{M_{1}+M_{2},R,N_{1}+N_{2}\right\}\left(\tau_{2}+\tau_{3}\right)/\left(\tau_{1}+\tau_{2}+\tau_{3}+\tau_{4}\right)$. According to the security and decoding analysis, the achievable sum-SDoF of proposed relay-aided jamming and two-receiver IA scheme is calculated through the following:

- If $N_{2}<R$, we substitute (17), (19), and (53) with equality or (59) with equality into $(\min\left\{M_{1}+M_{2},R,N_{1}+N_{2}\right\}\left(\tau_{2}+\tau_{3}\right)/\left(\tau_{1}+\tau_{2}+\tau_{3}+\tau_{4}\right)$ to derive the exact achievable sum-SDoF.
- If $N_{1}<R\leq N_{2}$, we substitute (17), $\tau_{2}=0$, and (59) with equality into $\min\left\{M_{1}+M_{2},R,N_{1}+N_{2}\right\}\left(\tau_{2}+\tau_{3}\right)/\left(\tau_{1}+\tau_{2}+\tau_{3}+\tau_{4}\right)$ to derive the exact achievable sum-SDoF.
- If $R\leq N_{1}$, we cannot ensure zero information leakage; hence, achievable sum-SDoF is zero.

The achievable sum-SDoF of proposed relay-aided jamming and two-receiver IA scheme is given in (4) for $N_{2}<M_{1}+M_{2}\;\;\&\;\;N_{2}<R$ Case, $N_{2}<M_{1}+M_{2}\;\;\&\;\;N_{1}<R\leq N_{2}$ Case, and $R\leq N_{1}$ Case.

## 5. Conclusions

We studied the sum-SDoF of the multiple-antenna full-duplex relay-aided MIMO XCCM with arbitrary antenna configurations, where there is no CSI at two transmitters and delayed CSI at the relay. To establish a sum-SDoF lower bound, we designed three transmission schemes. For the proposed schemes, full-duplex relay was utilized to receive the data symbol signals meanwhile transmit jamming signals, as this method can increase the transmission efficiency in contrast to half-duplex systems. We also derived a sum-SDoF upper bound. We characterized the sum-SDoF for 2N≤R and N≤M. We showed that the derived sum-SDoF lower bound can be higher than the sum-SDoF of the MIMO XCCM with output feedback and delayed CSIT for 2N≤R and M<N, which is not attained by the MIMO XCCM with no CSIT. Therefore, a multiple-antenna full-duplex relay with delayed CSI is beneficial for the MIMO XCCM with no CSIT from the SDoF perspective. In the future, there are several directions to extend this work, which are as follows: (1) a better sum-SDoF upper bound; (2) the impact of cognitive messages at transmitters/receivers; (3) MIMO X networks with more than two receivers; (4) the impact of imperfect self-interference cancellation on sum-SDoF characterization; and (5) the analysis of error performance of proposed schemes for finite SNR.

## Data Availability

Not applicable.

## Appendix A. The Rank of Matrices A_2_, B_2_

The matrix $A_{2}$ can be decomposed into the following:

$$\underset{A_{2,1}}{\underset{︸}{\left[\begin{array}{cc} I_{N_{1}\tau_{1}} & 0\\ H_{r,1}^{P\text{-}\mathrm{II}}\Phi_{1} & 0\\ 0 & I_{N_{1}\tau_{3}} \end{array}\right]}}\underset{A_{2,2}}{\underset{︸}{\left[\begin{array}{c} H_{r,1}^{P\text{-}I}\\ H_{r,1}^{P\text{-}\mathrm{III}}\Phi_{2}H_{r,2}^{P\text{-}I} \end{array}\right]}},$$

where the rank of matrix $A_{2,1}$ is $N_{1}\left(\tau_{1}+\tau_{3}\right)$ by Gaussian elimination, and the rank of matrix $A_{2,2}$ is $\min\{N_{1}\tau_{1}+\min\left\{R,N_{1}\right\}\tau_{3},\left(N_{1}+N_{2}\right)\tau_{1},R\tau_{1}\}$, due to $\mathrm{RK}\;H_{r,1}^{P\text{-}I}=\min\left\{N_{1},R\right\}\tau_{1}$, $\mathrm{RK}\;H_{r,2}^{P\text{-}I}=\min\left\{N_{2},R\right\}\tau_{1}$, $\mathrm{RK}\;\Phi_{2}=\min\left\{R\tau_{3},N_{2}\tau_{1}\right\}$, and $\mathrm{RK}\;H_{r,1}^{P\text{-}\mathrm{III}}=\min\left\{R,N_{1}\right\}\tau_{3}$. Therefore, the rank of matrix $A_{2}$ is $\min\{N_{1}\tau_{1}+\min\left\{R,N_{1}\right\}\tau_{3},\left(N_{1}+N_{2}\right)\tau_{1},R\tau_{1}\}$.

The matrix $B_{2}$ can be decomposed into the following:

$$\underset{B_{2,1}}{\underset{︸}{\left[\begin{array}{cc} I_{N_{2}\tau_{1}} & 0\\ 0 & I_{N_{2}\tau_{2}}\\ H_{r,2}^{P\text{-}\mathrm{III}}\Phi_{2} & 0 \end{array}\right]}}\underset{B_{2,2}}{\underset{︸}{\left[\begin{array}{c} H_{r,2}^{P\text{-}I}\\ H_{r,2}^{P\text{-}\mathrm{II}}\Phi_{1}H_{r,1}^{P\text{-}I} \end{array}\right]}},$$

where the rank of matrix $B_{2,1}$ is $N_{2}\left(\tau_{1}+\tau_{2}\right)$ by Gaussian elimination, and the rank of matrix $B_{2,2}$ is $\min\{N_{2}\tau_{1}+\min\left\{R,N_{2}\right\}\tau_{2},\left(N_{1}+N_{2}\right)\tau_{1},R\tau_{1}\}$, due to $\mathrm{RK}\;H_{r,2}^{P\text{-}I}=\min\left\{N_{2},R\right\}\tau_{1}$, $\mathrm{RK}\;H_{r,1}^{P\text{-}I}=\min\left\{N_{1},R\right\}\tau_{1}$, $\mathrm{RK}\;\Phi_{1}=\min\left\{R\tau_{2},N_{1}\tau_{1}\right\}$, and $\mathrm{RK}\;H_{r,2}^{P\text{-}\mathrm{II}}=\min\left\{R,N_{2}\right\}\tau_{2}$. Therefore, the rank of matrix $B_{2}$ is $\min\{N_{2}\tau_{1}+\min\left\{R,N_{2}\right\}\tau_{2},\left(N_{1}+N_{2}\right)\tau_{1},R\tau_{1}\}$.

## Appendix B. The Rank of Matrices H_1_

The matrix $H_{1}$ can be decomposed into the following:

$$\underset{H_{1,1}}{\underset{︸}{\left[\begin{array}{cc} I_{N_{1}\tau_{2}} & 0\\ 0 & H_{r,1}^{P\text{-}\mathrm{IV}}\Pi_{1} \end{array}\right]}}\underset{H_{1,2}}{\underset{︸}{\left[\begin{array}{cc} H_{1,1}^{P\text{-}\mathrm{II}} & H_{2,1}^{P\text{-}\mathrm{II}}\\ H_{1,2}^{P\text{-}\mathrm{II}} & H_{2,2}^{P\text{-}\mathrm{II}} \end{array}\right]}},$$

where the rank of matrix $H_{1,1}$ is $\min\{N_{1}\tau_{2}+\min\left\{N_{1},R\right\}\tau_{4},\left(N_{1}+N_{2}\right)\tau_{2}\}$, due to $\mathrm{RK}\;H_{r,1}^{P\text{-}\mathrm{IV}}=\min\left\{N_{1},R\right\}\tau_{4}$ and $\mathrm{RK}\;\Pi_{1}=\min\left\{N_{2}\tau_{2},\min\left\{N_{1},R\right\}\tau_{4}\right\}$, and the rank of matrix $H_{1,2}$ is $\min\{\left(N_{1}+N_{2}\right)\tau_{2},\left(M_{1}+M_{2}\right)\tau_{2},R\tau_{2}\}$, due to $\mathrm{RK}\;H_{1,j}^{P\text{-}\mathrm{II}}=\min\left\{N_{j},M_{1}\right\}\tau_{2},j=1,2$, and $\mathrm{RK}\;H_{2,j}^{P\text{-}\mathrm{II}}=\min\left\{M_{2},R-M_{1},N_{1}+N_{2}-M_{1},N_{j}\right\}\tau_{2},j=1,2$. Therefore, the rank of matrix $H_{1}$ follows $\min\{N_{1}\tau_{2}+\min\left\{N_{1},R\right\}\tau_{4},\left(N_{1}+N_{2}\right)\tau_{2},\left(M_{1}+M_{2}\right)\tau_{2},R\tau_{2}\}$. With $N_{1}<M_{1}+M_{2}\leq N_{2}$, the rank of matrix $H_{1}$ is $\min\{N_{1}\tau_{2}+\min\left\{N_{1}\tau_{4},R\tau_{4}\right\},\left(M_{1}+M_{2}\right)\tau_{2},R\tau_{2}\}$.

## Appendix C. The Rank of Matrices H_1_′ and H_2_′

The rank of matrix $H_{1}^{\prime }$ can be decomposed into the following:

$$\underset{H_{1,1}^{\prime }}{\underset{︸}{\left[\begin{array}{cc} I_{N_{1}\tau_{2}} & 0\\ 0 & H_{r,1}^{P\text{-}\mathrm{IV}}\Pi_{1} \end{array}\right]}}\underset{H_{1,2}^{\prime }}{\underset{︸}{\left[\begin{array}{cc} H_{1,1}^{P\text{-}\mathrm{II}} & H_{2,1}^{P\text{-}\mathrm{II}}\\ H_{1,2}^{P\text{-}\mathrm{II}} & H_{2,2}^{P\text{-}\mathrm{II}} \end{array}\right]}},$$

where the rank of matrix $H_{1,1}^{\prime }$ is $\min\{\min\left\{N_{1},R\right\}\tau_{4},\left(N_{1}+N_{2}\right)\tau_{2}\}$, due to $\mathrm{RK}\;H_{r,1}^{P\text{-}\mathrm{IV}}=\min\left\{N_{1},R\right\}\tau_{4}$ and $\mathrm{RK}\;\Pi_{1}=\min\left\{N_{2}\tau_{2},\min\left\{N_{1},R\right\}\tau_{4}\right\}$, and the rank of matrix $H_{1,2}^{\prime }$ is $\min\{\left(N_{1}+N_{2}\right)\tau_{2},\left(M_{1}+M_{2}\right)\tau_{2},R\tau_{2}\}$, due to $\mathrm{RK}\;H_{1,j}^{P\text{-}\mathrm{II}}=\min\left\{N_{j},M_{1}\right\}\tau_{2},j=1,2$, and $\mathrm{RK}\;H_{2,j}^{P\text{-}\mathrm{II}}=\min\left\{M_{2},R-M_{1},N_{1}+N_{2}-M_{1},N_{j}\right\}\tau_{2},j=1,2$. Therefore, the rank of matrix $H_{1}^{\prime }$ is $\min\{N_{1}\tau_{2}+\min\left\{N_{1},R\right\}\tau_{4},\left(N_{1}+N_{2}\right)\tau_{2},\left(M_{1}+M_{2}\right)\tau_{2},R\tau_{2}\}$.

The rank of matrix $H_{2}^{\prime }$ can be decomposed into the following:

$$\underset{H_{2,1}^{\prime }}{\underset{︸}{\left[\begin{array}{cc} I_{N_{2}\tau_{3}} & 0\\ 0 & H_{r,2}^{P\text{-}\mathrm{IV}}\Pi_{2} \end{array}\right]}}\underset{H_{2,2}^{\prime }}{\underset{︸}{\left[\begin{array}{cc} H_{1,1}^{P\text{-}\mathrm{III}} & H_{2,1}^{P\text{-}\mathrm{III}}\\ H_{1,2}^{P\text{-}\mathrm{III}} & H_{2,2}^{P\text{-}\mathrm{III}} \end{array}\right]}},$$

where the rank of matrix $H_{2,1}^{\prime }$ is $\min\{\min\left\{N_{1},R\right\}\tau_{4},\left(N_{1}+N_{2}\right)\tau_{3}\}$, due to $\mathrm{RK}\;H_{r,2}^{P\text{-}\mathrm{IV}}=\min\left\{N_{1},R\right\}\tau_{4}$ and $\mathrm{RK}\;\Pi_{2}=\min\left\{N_{1}\tau_{3},\min\left\{N_{1},R\right\}\tau_{4}\right\}$, and the rank of matrix $H_{2,2}^{\prime }$ is $\min\{\left(N_{1}+N_{2}\right)\tau_{3},\left(M_{1}+M_{2}\right)\tau_{3},R\tau_{3}\}$, due to $\mathrm{RK}\;H_{1,j}^{P\text{-}\mathrm{III}}=\min\left\{N_{j},M_{1}\right\}\tau_{3},j=1,2$, and $\mathrm{RK}\;H_{2,j}^{P\text{-}\mathrm{III}}=\min\left\{M_{2},R-M_{1},N_{1}+N_{2}-M_{1},N_{j}\right\}\tau_{3},j=1,2$. Therefore, the rank of matrix $H_{2}^{\prime }$ is $\min\{N_{2}\tau_{3}+\min\left\{N_{1},R\right\}\tau_{4},\left(N_{1}+N_{2}\right)\tau_{3},\left(M_{1}+M_{2}\right)\tau_{3},R\tau_{3}\}$.
